# Association of childhood infections and perinatal factors with ankylosing spondylitis: a Swedish nationwide case–control and sibling study

**DOI:** 10.1136/rmdopen-2023-003438

**Published:** 2023-10-16

**Authors:** Matilda Morin, Karin Hellgren, Ulf Lindström, Thomas Frisell

**Affiliations:** 1 Clinical Epidemiology Division, Department of Medicine, Solna, Karolinska Institutet, Stockholm, Sweden; 2 Department of Rheumatology and Inflammation Research, Sahlgrenska Academy at University of Gothenburg, Gothenburg, Sweden

**Keywords:** spondylitis, ankylosing, epidemiology

## Abstract

**Objectives:**

To identify perinatal and early-life risk factors for ankylosing spondylitis (AS), controlling for family-shared confounding with a sibling comparison design.

**Methods:**

In this nationwide, register-based case–control study, we identified 5612 AS cases from the Swedish National Patient Register, and matched them with 22 042 individuals without inflammatory arthritis from the general population. Conditional logistic regression was used to estimate odds ratios (ORs) and 95% confidence intervals (CIs) of AS in relation to childhood infections and a broad range of perinatal factors including fetal growth. Significant associations were further tested in a sibling comparison analysis, including 3965 patients with AS and their 6070 siblings without a diagnosis of spondyloarthritis.

**Results:**

We found no statistically significant associations between any studied fetal growth-related factor or other perinatal factors and the risk of developing AS. In contrast, having older siblings (adjusted OR 1.12; 95% CI 1.04 to 1.22 for one vs no older sibling) and history of a childhood tonsillectomy (adjusted OR 1.30; 95% CI 1.13 to 1.49) were associated with AS in the case–control analysis, results that also held in the sibling comparison. Serious childhood infection and multiple birth were significantly associated with AS in the case–control sample, but estimates were attenuated in the sibling comparison.

**Conclusions:**

Having older siblings and a history of tonsillectomy in childhood were independently associated with development of AS, even after adjustment for family-shared factors in a sibling comparison analysis. This strengthens the hypothesis that childhood infections play a role in the aetiology of AS.

**WHAT IS ALREADY KNOWN ON THIS TOPIC**

- Environmental exposures during the perinatal period and early life have been suggested to increase the risk of chronic inflammatory diseases.
- Specifically for ankylosing spondylitis (AS), some studies reported season of birth, birth order among siblings and childhood infections to be associated with disease development in adulthood.
- A concern when studying perinatal and early-life exposures in relation to adult disease is the risk of confounding from, for example, socioeconomic conditions in childhood.

**WHAT THIS STUDY ADDS**

- In this study, with prospectively collected data from national registers, we found that having older siblings and a history of tonsillectomy in childhood were associated with an increased risk of AS.
- These results remained after adjustment for family-shared confounders with a sibling comparison design.

**HOW THIS STUDY MIGHT AFFECT RESEARCH, PRACTICE OR POLICY**

- Despite the strong heritability in AS, environmental factors and particularly infections deserve continued research focus, to elucidate the mechanisms by which they contribute to AS development.

## Introduction

Ankylosing spondylitis (AS) is a chronic inflammatory arthritis within the spondyloarthritis (SpA) group, characterised by inflammatory back pain and radiographic changes to the sacroiliac joints.^1^ With an estimated heritability of 77%,^2^ genetic predisposition plays a vital role in the aetiology of AS. The most prominent risk allele is HLA-B27, but many more loci have been associated with AS in genome-wide association studies.^3 4^ Regarding environmental factors, which consequently explain at least 20% of variance in the liability to develop AS, current theories involve mechanical stress,^5 6^ infections^7 8^ or alterations of the gut microbiome,^9–11^ but the mechanisms by which these factors contribute to pathogenesis are not fully understood.

Studies with conflicting results have also linked factors related to the perinatal period and early life to chronic inflammatory diseases. These studies were motivated by a general interest in the possible developmental origins of adult disease, which was sparked by the finding of an inverse association between birth weight and adult coronary heart disease in the 1980s.^12 13^ While the first theories focused on maternal nutrition, other early-life events have also been suggested to alter adult disease risk—by affecting the maturing immune system.^14^ Caesarean delivery, birth weight and having older siblings have been associated with future rheumatoid arthritis, although without conclusive evidence.^15–18^ Caesarean delivery has also been associated with inflammatory bowel disease (IBD).^19 20^ In AS, birth order among siblings and season of birth have been associated with adult disease, but most studies have been small and findings have often proved difficult to replicate.^21–25^ Childhood hospitalisations for respiratory tract infections have also been associated with AS,^8^ while childhood appendicitis has been reported to be protective for both AS and IBD.^8 26 27^

Studies of perinatal risk factors for adult disease can be susceptible to bias due to poor validity in exposure measures. The most influential previous studies have relied on prospectively recorded data in national birth registers. Owing to the long follow-up needed from birth to disease onset, these studies have so far been limited by relatively small samples. Further, even with high validity measures, a remaining problem when studying early-life exposures is the risk of confounding, either from genetic factors (which are strong for AS), or other factors associated with both AS and the early-life exposure—for example, maternal characteristics and childhood socioeconomic background. These confounding factors are difficult to measure and hence to adjust for in conventional analyses. An alternative approach is to study the association in siblings, which implicitly adjusts for confounding from (measured or unmeasured) environmental factors shared within families, such as maternal factors and early environment,^28^ but this design has to our knowledge not previously been used to evaluate risk factors for AS.

Therefore, we used Swedish nationwide population-based registers to perform the hitherto most well-powered investigation of the association of childhood infections and a broad range of perinatal factors, including fetal growth, with onset of AS in adulthood. We also, for the first time in a study of SpA, used a sibling comparison design including the patients’ siblings without a SpA diagnosis to adjust associations for confounding from factors shared by siblings.

## Methods

### Study design

This nationwide register-based case–control study compared the exposure to perinatal factors and childhood infections in cases with AS and general-population controls. In accordance with a predefined study plan, statistically significant associations were then tested in a sibling comparison analysis, including AS cases and their siblings without a diagnosis of SpA, to control for family-shared confounding.

### Study population

From the Swedish National Patient Register (NPR), we identified AS cases as individuals with at least one inpatient or outpatient visit with a recorded diagnosis of AS (International Classification of Diseases, 10th edition codes M45.9 or M08.1) at a specialist clinic in rheumatology or internal medicine, between January 2001 and December 2022. All healthcare providers in Sweden are required by law to provide data to the NPR, on hospitalisations since 1964 (with complete nationwide coverage since 1987) and outpatient specialist visits since 2001.^29^ The validity of an AS diagnosis in the NPR is high, with a positive predictive value of 80% to fulfil the modified New York criteria.^30^ We also required the births of the cases to be registered in the Medical Birth Register (MBR), which includes over 98% of all births in Sweden since 1973.^31^ Each AS case was matched on sex, year of birth and region of residence to five control individuals from the general population, who did not have a diagnosis of chronic inflammatory arthritis at time of diagnosis in the index case. Controls whose births were not recorded in the MBR were excluded.

For the sibling comparison analysis, we identified full siblings of AS cases from the Multi-Generation Register, which link Swedish individuals with their parents, and therefore their siblings, via their personal identity numbers.^32^ Siblings were included in the analysis if their birth was registered in the MBR and they had no healthcare visit in the NPR with a recorded diagnosis of SpA (here defined as AS, psoriatic arthritis, enteropathic arthritis, reactive arthritis or undifferentiated SpA), IBD or psoriasis (detailed definition in online supplemental table 1) during the study period (2001–2022).

10.1136/rmdopen-2023-003438.supp1 Supplementary data

### Patient and public involvement

Patients or public representatives were not involved in the design or conduct of this study.

### Exposures and covariates

We included exposures from three main areas previously under consideration as early-life risk factors for AS: intrauterine environment, family circumstances and childhood infections. We identified perinatal exposures in the MBR, including maternal age at delivery, maternal smoking and BMI in early pregnancy, length of gestation, weight for gestational age (in SDs from sex-specific mean weight per gestational age), birth weight, multiple birth, Caesarean delivery, maternal infection during pregnancy and season of birth. We investigated the association with number of siblings, with total number of full siblings and younger siblings identified in the Multi-Generation Register, and number of older siblings determined from parity reported in the MBR. Additionally, serious childhood infections were identified as a registered infection in the MBR at birth or as a hospitalisation with an ICD code for infection during the first 15 years of life (ICD codes in online supplemental table 2). Based on previous research,^8^ we also selected the procedures tonsillectomy and appendectomy before age 16, identified in the NPR, as potential risk factors.

Covariates were selected to capture socioeconomic background and included maternal age, maternal BMI, maternal smoking, parental country of birth (both born in a Nordic country vs not, from the Total Population Register), maternal disposable income in the year before delivery, and educational level in the year of delivery (from the Longitudinal Integrated Database for Health Insurance and Labour Market Studies (LISA) and national censuses). We also included multiple birth and an indicator for maternal inflammatory disease any time before delivery, defined as a hospitalisation in the NPR with a diagnosis of SpA, IBD or psoriasis (definitions in online supplemental table 1).

### Statistical analysis

We used separate conditional logistic regression models in the matched case–control data to estimate crude and adjusted odds ratios (ORs) and 95% confidence intervals (CIs) for AS in relation to each exposure. Maternal BMI, gestational age, weight for gestational age and birth weight were analysed both as continuous exposures (as second-degree polynomials) and in categories, in order to catch associations within specific ranges of these variables. Analyses were adjusted for the above-mentioned covariates, with the exception that no exposure was adjusted for itself. Number of older siblings was additionally adjusted for number of younger siblings, and vice versa. The analyses for maternal smoking and BMI were restricted to births from year 1982 onwards, when this information was first available in the MBR. To investigate potential effect modification by sex, we also conducted the same analyses stratified by sex.

The proportion of missing data was low for most variables, but higher for maternal smoking and BMI, with no data available for births before 1982. The proportion of missing data per variable is found in online supplemental table 3. We used multiple imputation to account for missing data in the case–control analysis. Fully conditional specification was used to create 25 imputed datasets, including the outcome, all exposure variables and covariates. Continuous variables were included both as second-degree polynomials, imputed with predictive mean matching, and in categories. For comparison, we also conducted the analysis among individuals with complete data on all variables.

Analyses were performed in SAS version 9.4.

### Sibling comparison

In the sibling comparison analysis, exposures with statistically significant associations with AS in the case–control analysis were analysed with logistic regression conditioning on the family. Each cluster consisted of one AS case and all of his or her siblings without a diagnosis of SpA whose births were registered in the MBR (ie, born in Sweden from 1973). By design, only siblings who are discordant for both exposure and outcome contribute to the regression estimates. While the sibling analysis inherently adjusts for childhood socioeconomic factors, we adjusted for factors that can vary between siblings: maternal age at delivery, year of birth, sex of child and parity (except when exposure was number of siblings). For the variables included in the sibling comparison analysis, there were no missing data.

### Sensitivity analyses

To verify the validity of the sibling comparison, we conducted several sensitivity analyses. To assess the impact of left truncation (ie, that subjects born in later years had less time to be registered with AS), we restricted analyses to cases and siblings who reached at least 35 years of age before the end of follow-up (December 2022). We also did the same restriction in the analysis of cases and population controls. Additionally, we examined if individuals who had siblings differed from the full population by restricting the case–control analysis to cases and controls with at least one sibling.

## Results

We identified 6771 individuals born 1973 onwards who had an AS diagnosis in the NPR between 2001 and 2022. Of these, 5612 (43% females) were born in Sweden with births registered in the MBR and they were selected as cases. Each case was matched to five general population controls, of whom 22 042 had their birth registered in the MBR, resulting in an average number of four controls per case. AS cases and population controls were comparable on background characteristics such as maternal age at delivery and proportion with both parents born in a Nordic country (table 1). Having a mother hospitalised for SpA, IBD or psoriasis was more common for cases (0.7% vs 0.4%), as would be expected considering the familiality of these diseases. Among AS cases, 27% had a registered diagnosis of anterior uveitis in the NPR, 7% of psoriasis, and 8% of IBD.

**Table 1:** Background characteristics of AS cases and population controls, matched on sex, year of birth, and region of residence

Characteristics	AS cases (n=5612)	Population controls (n=22 042)
Female, n (%)	2411 (43.0)	9450 (42.9)
Year of birth, n (%)		
1973–1979	2006 (35.7)	7838 (35.6)
1980–1989	2285 (40.7)	8799 (39.9)
1990–1999	1176 (21.0)	4833 (21.9)
2000–2004	145 (2.6)	572 (2.6)
SpA manifestations*, n (%)		
Anterior uveitis	1521 (27.1)	N/A
Psoriasis	396 (7.1)	N/A
IBD	420 (7.5)	N/A
Both parents born in Nordic country, n (%)	5116 (91.7)	19 931 (91.0)
Maternal age at delivery in years, mean (SD)	27.4 (5.0)	27.5 (5.1)
Maternal educational level†, n (%)		
≤9 years	1221 (22.0)	5011 (23.1)
10–12 years	2805 (50.6)	11 048 (50.9)
>12 years	1515 (27.3)	5656 (26.0)
Maternal inflammatory disease‡	42 (0.7)	79 (0.4)

Proportions among those with available data for each variable. Proportion of missing data per variable in online supplemental table 3.

*As registered in the National Patient Register 1997–2022. Only assessed in AS cases.

†In year of delivery.

‡Hospitalisation for SpA, IBD, or psoriasis ever before delivery.

AS, ankylosing spondylitis; IBD, inflammatory bowel disease; SpA, spondyloarthritis.

### Odds ratios for AS comparing cases and population controls

**Figure 1:**
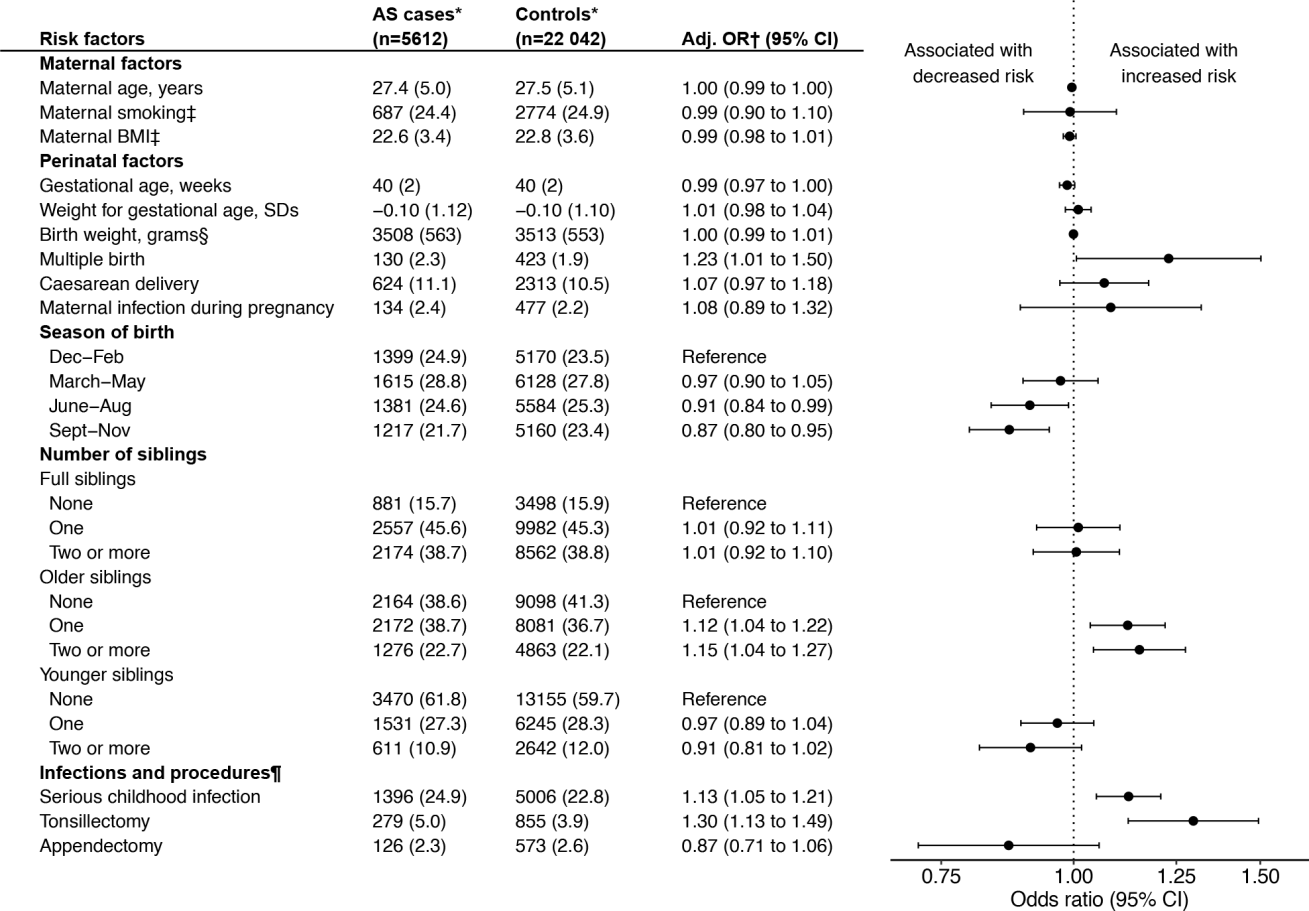
ORs of AS in relation to childhood infections and perinatal factors. ORs from conditional logistic regression on 25 multiply imputed datasets comparing AS cases and population controls, matched on sex, year of birth and region of residence. Unadjusted estimates and results for categorical versions of continuous exposure variables in online supplemental table 4. *Numbers are n (%) or mean (SD). †Adjusted for maternal age, maternal BMI, maternal smoking, parental country of birth, maternal disposable income, maternal educational level, maternal inflammatory disease (hospitalisation for SpA, IBD, psoriasis), and multiple birth, with the exception that no exposure was adjusted for itself. Number of older siblings additionally adjusted for number of younger siblings, and vice versa. ‡Analyses for smoking and BMI restricted to birth years from 1982 onwards. §Analysed per 100 g. ¶Until age 15. Sixteen cases and their 72 matched controls excluded owing to AS diagnosis in index case before age 16. AS, ankylosing spondylitis; BMI, body-mass index; IBD, inflammatory bowel disease; SpA, spondyloarthritis.

Comparing exposures between AS cases and controls, we found a number of exposures significantly associated with AS in the multiply imputed data (figure 1). Having older siblings, but not siblings in general, was a risk factor for AS, with similar ORs for having just one or more older siblings. Significant associations with AS were found for both serious childhood infections (adjusted OR 1.13; 95% CI 1.05 to 1.21) and tonsillectomy before age 16 (adjusted OR 1.30; 95% CI 1.13 to 1.49). Multiple birth, in contrast to singleton birth, was associated with AS (adjusted OR 1.23; 95% OR 1.01 to 1.50). Being born in the summer or autumn months was associated with a significantly lower risk of AS compared with being born in winter. History of an appendectomy before age 16 was associated with a non-significantly reduced AS risk (adjusted OR 0.87; 95% CI 0.71 to 1.06). We found no statistically significant associations with birth weight, maternal smoking or other maternal or perinatal factors, though elevated point estimates were noted for very preterm birth, small for gestational age birth, Caesarean delivery, and maternal infection during pregnancy. For unadjusted estimates and results for categorical versions of continuous exposure variables, see online supplemental table 4.

In the complete-case analysis, which excluded everyone born before 1982 owing to lack of data on maternal BMI and smoking in early pregnancy, results were similar to those from multiple imputation for most exposures (online supplemental table 5). For maternal BMI, both high (≥30 kg/m^2^) and low (<18.5 kg/m^2^) BMI resulted in statistically significantly reduced odds for AS (adjusted OR 0.75; 95% CI 0.58 to 0.96 for underweight and 0.70; 95% 0.52 to 0.94 for obesity). A weight for gestational age 1–2 SDs above the sex-specific mean weight for gestational age, but not >2 SDs above, resulted in an increased OR for AS (adjusted OR 1.24; 95% CI 1.05 to 1.46).

### Differences in men and women

**Figure 2:**
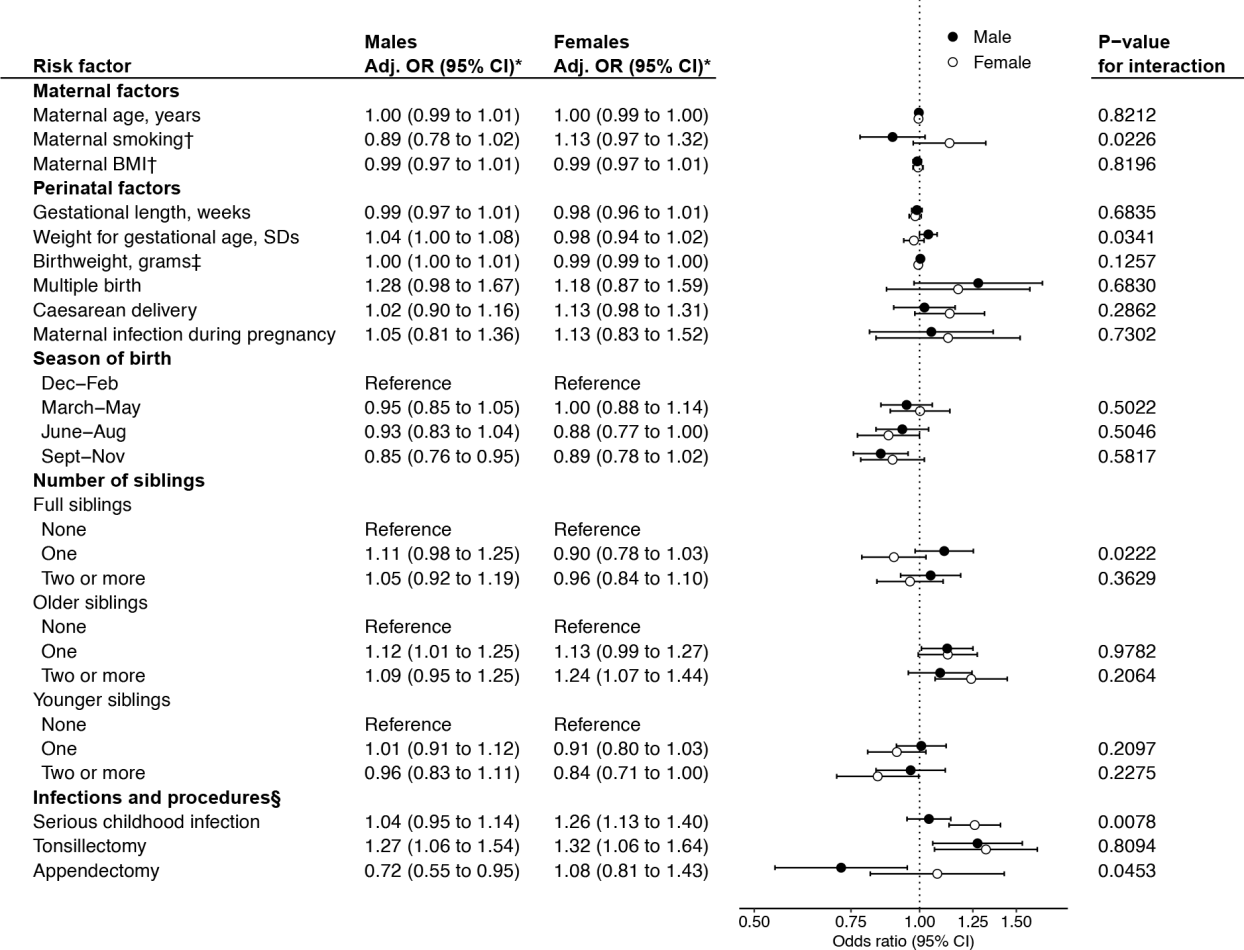
ORs for AS in males and females separately, in relation to childhood infections and perinatal factors, with p values for significance of interaction by sex. ORs from conditional logistic regression on 25 multiply imputed datasets comparing AS cases and population controls, matched on sex, year of birth and region of residence. Number and proportion with each exposure, unadjusted estimates and categorical versions of continuous exposure variables in online supplemental table 6. *Adjusted for maternal age, maternal BMI, maternal smoking, parental country of birth, maternal disposable income, maternal educational level, maternal inflammatory disease (hospitalisation for SpA, IBD, psoriasis), and multiple birth, with the exception that no exposure was adjusted for itself. Number of older siblings additionally adjusted for number of younger siblings, and vice versa. †Analyses for smoking and BMI restricted to birth years from 1982 onwards. ‡Analysed per 100 g. §Until age 15. AS, ankylosing spondylitis; BMI, body-mass index; IBD, inflammatory bowel disease; SpA, spondyloarthritis.

Stratifying the analysis by sex resulted in few significant associations (figure 2). Significant effect modification by sex, however, was seen for several exposures. Most notably, serious childhood infection was a risk factor for AS in women but not among men (p for interaction=0.0078), and in contrast, the seemingly protective effect of appendectomy was present in men only (p=0.045). Number and proportion with each exposure, unadjusted estimates, and categorical versions of continuous exposure variables are shown in online supplemental table 6.

### Odds ratios for AS from sibling comparison analysis

For the sibling comparison analysis, we were able to include 3965 AS cases and their 6070 SpA-free siblings (606 siblings excluded owing to having received a diagnosis of SpA themselves). In the analysis of exposure-discordant and outcome-discordant siblings, the association between multiple birth and AS observed in the case–control analysis was reduced to OR 1.09; 95% CI 0.75 to 1.58 (table 2). We found no association between season of birth and AS among siblings. The risk associated with having older siblings, on the other hand, was more pronounced in the sibling comparison, at least after adjustment for sex, maternal age and year of birth (OR 1.18; 95% CI 1.06 to 1.31 for having one older sibling vs none, and OR 1.34; 95% CI 1.09 to 1.65 for having two or more older siblings). The association with serious childhood infections observed in the case–control analysis was attenuated in the sibling comparison (adjusted OR 1.04; 95% CI 0.94 to 1.16), but the association with tonsillectomy was stronger among siblings (adjusted OR 1.36; 95% CI 1.10 to 1.67).

**Table 2:** ORs for AS from sibling comparison analysis

Risk factor	AS cases* (n=3965)	SpA-free siblings* (n=6070)	OR (95% CI)	Adj. OR† (95% CI)
Multiple birth, n (%)	118 (3.0)	208 (3.4)	1.06 (0.73 to 1.52)	1.09 (0.75 to 1.58)
Season of birth, n (%)				
Dec-Feb	979 (24.7)	1461 (24.1)	Reference	Reference
March-May	1136 (28.7)	1725 (28.4)	0.99 (0.88 to 1.10)	0.99 (0.89 to 1.12)
June-Aug	978 (24.7)	1517 (25.0)	0.97 (0.86 to 1.09)	0.96 (0.85 to 1.08)
Sep-Nov	872 (22.0)	1367 (22.5)	0.99 (0.87 to 1.12)	0.96 (0.84 to 1.10)
No. of older siblings, n (%)				
0	1462 (36.9)	1836 (30.2)	Reference	Reference
1	1589 (40.1)	2089 (34.4)	0.96 (0.89 to 1.03)	1.18 (1.06 to 1.31)
2 or more	914 (23.1)	2145 (35.3)	0.78 (0.70 to 0.86)	1.34 (1.09 to 1.65)
Serious childhood infection‡, n (%)	1006 (25.5)	1503 (24.8)	1.05 (0.95 to 1.16)	1.04 (0.94 to 1.16)
Tonsillectomy‡, n (%)	210 (5.3)	253 (4.2)	1.37 (1.12 to 1.68)	1.36 (1.10 to 1.67)

ORs from conditional logistic regression.

*Numbers are n (%). Numbers represent outcome-discordant siblings. Only siblings discordant for both exposure and outcome contribute to the regression estimates.

†Adjusted for maternal age, year of birth, sex of child and parity (except when exposure is number of older siblings).

‡Until age 15. Fourteen cases and their 18 SpA-free siblings excluded owing to AS diagnosis in index case before age 16.

AS, ankylosing spondylitis; SpA, spondyloarthritis.

### Sensitivity analyses

To address the impact of left truncation, we restricted the sibling analysis to individuals who reached at least 35 years of age before the end of follow-up (online supplemental table 7). In this group, we observed even stronger associations with number of older siblings (adjusted OR 1.35; 95% CI 1.16 to 1.57 for one older sibling, and OR 1.75; 95% CI 1.31 to 2.34 for two or more), and tonsillectomy (adjusted OR 1.44; 95% CI 1.06 to 1.96). We also noted a higher point estimate for the association with multiple birth (adjusted OR 1.36; 95% CI 0.79 to 2.33). Similar restriction in the case–control analysis also resulted in a stronger association with multiple birth (adjusted OR 1.51; 95% CI 1.18 to 1.92) (online supplemental table 8).

Restricting the case–control analysis to individuals with siblings resulted in similar associations as in the full sample (online supplemental table 9). This indicates that the difference of estimates between the case–control and sibling analyses is not a consequence of individuals with siblings being different from singletons in critical ways.

### Post hoc analyses

To investigate whether timing of infections and infection-related procedures mattered, we performed a number of post hoc analyses. Restricting to serious childhood infections during the first year of life (instead of first 15 years) gave a similar point estimate in the case–control data, although no longer significant (online supplemental table 10). Stratifying the analyses for tonsillectomy and appendectomy by age at procedure (<5 years, 5–9 years, 10–15 years), associations were strongest for procedures between 5 and 9 years of age.

We also evaluated an alternative definition for appendectomy, only including individuals with codes for both appendicitis and appendectomy in the NPR (85 (1.5%) of cases and 469 (2.1%) of controls). This resulted in an OR for AS of 0.72 (95% CI 0.57 to 0.91).

## Discussion

In this nationwide study of a large cohort of AS cases and general population controls, we identified early-life risk factors for AS from prospectively collected register data. We also, for the first time in a study of SpA, validated those results in a sibling comparison analysis, to control for confounding factors shared within families.

We found that the risk of AS increased with the number of older siblings. This result also held in the sibling comparison, suggesting that the association is not a result of confounding from familial factors. The mechanism behind this risk increase cannot be determined from our data, but it has been shown that infants with older siblings are more exposed to infections early in life than infants without siblings.^33 34^ Interestingly, we also found serious childhood infections and especially tonsillectomy in childhood to be associated with AS. While the association with serious childhood infection was attenuated in the sibling comparison, the association with tonsillectomy remained.

This is the second study of perinatal risk factors for AS using Swedish national registers, and while we could increase the sample size by almost 300%, study a broader range of risk factors and include a sibling comparison, our findings remain in line with this initial publication.^24^ The previous study reported an OR for AS among those with older siblings very similar to ours (OR 1.18; 95% CI 1.06 to 1.30) and found a non-significantly increased risk associated with multiple birth (1.35; 95% CI 0.95 to 1.90). The significant OR for multiple birth from our case–control analysis, however, was largely attenuated in the sibling comparison.

A previous study of childhood infections and AS using Swedish data did not find a significant association between childhood hospitalisation with any infection and adult AS (OR 1.08; 95 % CI 0.96 to 1.22).^8^ Proportions of infections in both cases and controls in that study were lower (17.4% and 16.3%, respectively) compared with our current findings, possibly due to differing definitions. Our definition of serious infection is based on a more comprehensive list of ICD codes related to infections, and also includes infections recorded in the MBR at birth. The significant association from our case–control analysis was attenuated in the sibling comparison, but this attenuation does not necessarily indicate that the population estimate is confounded. The choice of serious infection as an exposure, compared with any infection, is a consequence of data availability. The NPR contains only inpatient data during the childhood years of most study participants, but hospitalisation does not reflect the total burden of infections, which might be more important. Under that scenario, if two siblings had the same infection but only one required hospital care, the association in the sibling analysis will be attenuated; a special case of a known feature of sibling comparison studies where the presence of random measurement error in the exposure variable will attenuate the estimate from discordant sibling pairs more than the estimate in the full population.^35^

This is in line with the finding of an association with tonsillectomy, where repeated infections are probable to lead up to the procedure. As tonsillectomy had been associated with particularly strong odds for AS in the previous study using Swedish data,^8^ we included this procedure in our analysis to be able to test the association for family-shared confounding, and concluded that the result held in the sibling comparison. We found the highest OR for tonsillectomies performed between 5 and 9 years of age, but an association between adult tonsillitis and AS has also been reported, with a stronger association for longer interval between the two diagnoses.^7^ Interestingly, in a large population-based study of IBD in Denmark, associations were found between tonsillectomy (at any age) and IBD, and also with tonsillectomy in a relative and IBD.^36^ This would suggest that the association between tonsillectomy and IBD is caused, at least in part, by genetic or environmental factors shared within families, a result that could seemingly contradict our conclusions regarding tonsillectomy and AS. Genetic overlap between AS and IBD has been confirmed in several studies.^3 4 37^ That the association remained in discordant sibling pairs in our analysis is, however, clear evidence that family-shared factors cannot be the main explanation for the association between tonsillectomy and AS.

How childhood infections might lead to AS can have several possible explanations. Early infections could affect the maturation of the immune system, which continues after birth.^38^ However, restricting the analysis to serious infections during the first year of life did not increase the observed association as might have been expected if this was the main explanation. Treatment with antibiotics could alter the gut microflora, which is already suspected to be involved in AS development.^10 11^ Unfortunately, data on antibiotic prescriptions in Sweden are only available from mid-2005 onwards, so most individuals in our cohort will not have data on antibiotic prescriptions during childhood.

Significant associations with birth season have been reported earlier, in a Korean study of AS cases and population comparators, where being born in winter conferred the highest risk, and being born in spring the lowest.^23^ The association between season of birth and AS in our case–control analysis was almost entirely attenuated in the sibling comparison, suggesting influence of familial factors. It is not immediately obvious what these familial factors are, but there may be a demographic gradient in timing of birth that is also associated with some other risk factor to develop AS.

Childhood appendicitis with appendectomy has previously been associated with both a lower risk of AS,^8^ and a lower risk of IBD, especially ulcerative colitis.^26 27^ While we found a point estimate suggesting a protective effect of appendectomy (with or without appendicitis) in AS, it was less pronounced than in previous studies and failed to reach statistical significance. In the post hoc analysis where we included only appendectomies among individuals also diagnosed with appendicitis, we found a significant association, but with a smaller effect than in the previous Swedish study.^8^ In our complete-case analysis, which included only births from 1982 onwards, the protective association totally disappeared. When stratified by sex, the association remained only among males.

Among the three main areas of early-life exposures under consideration, intrauterine environment stood out by not displaying any significant associations with AS. Fetal growth has been associated with rheumatoid arthritis in several studies,^15–18^ but we did not find any growth-related factor associated with AS despite our sample size.

While the sibling comparison design is an ingenious way to adjust for shared environmental factors, it also comes with some limitations. The fact that only exposure-discordant and outcome-discordant siblings contribute to regression estimates reduces power, but this was not a major issue in this study. AS is a relatively uncommon condition even among relatives of patients with AS, and none of the studied exposures (or categories of them) was present in more than 40% of study subjects, or expected to cluster in families to any large extent. Another issue specific to the sibling comparison design is that it might amplify confounding from factors not shared by siblings, due to the selection of discordant sibling pairs,^35^ but for the same reasons as stated above we do not believe this to be a problem in this study. Random measurement error will also attenuate associations more in a sibling comparison than in a population sample.^35^ This might be the case for serious childhood infection, as discussed above, but for exposures registered in the MBR—for example, gestational age and Caesarean delivery, we would not expect measurement error to be a large problem.

Other limitations of the study include that we had access to outpatient data in the NPR only from 2001, and no data from general practitioners, thus having to restrict our definitions of infectious exposures to infections requiring hospitalisation and hard procedures like tonsillectomy. Additionally, we could not study the effect of antibiotic exposure, as we did not have access to prescription data before 2005. Another limitation is that the MBR contains only births from 1973, so that the oldest in our AS cohort were 49 and the youngest 18 at end of follow-up. This limits the number of AS cases available for inclusion, but also increases the risk that siblings who appear free of SpA might yet receive a diagnosis. We addressed this in a sensitivity analysis by restricting the sibling analysis to individuals who reached 35 years of age before the end of follow-up (ie, those born before 1986), which further strengthened the associations with older siblings and tonsillectomy.

A major strength of this case–control study, the largest to date on early-life risk factors for AS, is the use of national registers with virtually complete coverage, which enabled the inclusion of population controls, long follow-up and no recall bias for perinatal factors. Additionally, this is to our knowledge the first study to use a sibling comparison design to study early-life risk factors for AS, factors that are otherwise at high risk of family-level confounding.

## Conclusion

We found that having older siblings and a history of tonsillectomy in childhood were independently associated with development of AS, even after adjustment for family-shared factors in a sibling comparison analysis. Despite that the association with hospitalised infection was attenuated in the sibling comparison, our findings strengthen the hypothesis that childhood infections play a role in the aetiology of AS.

## Data Availability

Data may be obtained from a third party and are not publicly available.
