# Supplementary material for: Association of childhood infections and perinatal factors with ankylosing spondylitis: a Swedish nationwide case–control and sibling study
Source: RMD Open. 2023 Oct 16;9(3):e003438. doi: 10.1136/rmdopen-2023-003438 (PMC10582879; doi:10.1136/rmdopen-2023-003438)
Supplement: Supplementary data [file rmdopen-2023-003438supp001.pdf]

SUPPLEMENTAL MATERIAL

Association of childhood infections and perinatal factors with ankylosing spondylitis: a Swedish nationwide case-control and sibling study

Matilda Morin, MSc[1], Karin Hellgren, MD, PhD[1], Ulf Lindström, MD, PhD[2], Thomas Frisell, PhD[1]

[1] Clinical Epidemiology Division, Department of Medicine, Solna, Karolinska Institutet, Stockholm, Sweden
[2] Department of Rheumatology and Inflammation Research, Sahlgrenska Academy, University of Gothenburg, Gothenburg, Sweden

Supplemental table 1. Sources and definitions of outcome, exposures, and covariates

	Source	ATC and ICD-10 codes	Details/Definition
**Outcome**			
Ankylosing spondylitis	NPR	ICD-10 M45.9, M08.1	≥1 visit at specialist clinic of internal medicine or rheumatology, with a recorded diagnosis of AS after 2001.
**Exposures**			
Maternal age	MBR		In years. Modelled as second-degree polynomial.
Maternal smoking	MBR		At first antenatal visit. Coded smoker/non-smoker.
Maternal BMI	MBR		In kg/m^2, at first antenatal visit. Modelled as second-degree polynomial, and in categories <18.5, 18.5-24.9, 25-29.9, ≥30.
Gestational age	MBR		In weeks. Modelled as second-degree polynomial, and in categories very preterm (<32 weeks), preterm (32-36 weeks), term (37-40 weeks), post-term (≥41 weeks).
Weight for gestational age	MBR		In standard deviations from sex-specific mean weight per gestational age in growth curves by Marsál et al. Modelled as second-degree polynomial and in categories small for gestational age (SGA; >2 SD below), moderately small (1-2 SD below), normal (±1 SD), moderately large (1-2 SD above), and large for gestational age (LGA; >2 SD above).
Birth weight	MBR		Modelled as second-degree polynomial per 100 g, and in categories <2500 g, 2500-4299 g, ≥4300 g.
Multiple birth	MBR		Coded singleton/multiple.
Caesarean delivery	MBR		Including both elective and emergency Caesarean delivery.
Maternal infection during pregnancy	NPR, MBR	See Supplemental table 2	Hospitalisation with ICD code for infection during pregnancy, or infection recorded in MBR. Where data on gestational age was missing, it was assumed to be 40 weeks.
Season of birth	MBR		Coded winter (December-February), spring (March-May), summer (June-August), autumn (September-November).
Number of full siblings	MGR		Coded 0, 1, ≥2.
Number of older siblings	MBR		Based on parity variable in MBR. Coded 0, 1, ≥2.
Number of younger siblings	MGR		Only including younger full siblings. Coded 0, 1, ≥2.
Serious childhood infection	NPR, MBR	See Supplemental table 2	Infection recorded at birth (in MBR) or hospitalisation with ICD code for infection as main diagnosis between ages 0-15 (excluding index patients with AS diagnosis before age 16).

Cont. from previous page	Source	ATC and ICD-10 codes	Details/Definition
Tonsillectomy	NPR	Procedure codes 2710, 2720, 2730 (1973-1996) or EMB10, EMB15, EMB20, EMB30 (1997-)	Between ages 0-15 (excluding index patients with AS diagnosis before age 16).
Appendectomy	NPR	Procedure codes 4510, 4511, 0058 (1973-1996) or JEA00, JEA01, JEA10 (1997-)	Between ages 0-15 (excluding index patients with AS diagnosis before age 16).
Appendicitis with appendectomy	NPR	ICD-8 540-543<br>ICD-9 540-543<br>ICD-10 K35-38<br>plus procedure code for appendectomy (see above)	Both appendicitis and appendectomy recorded in NPR between ages 0-15 (excluding index patients with AS diagnosis before age 16).
**Other covariates**			
Parental country of birth	Total Population Register		Coded "Both born in Nordic country" vs. not
Maternal disposable income	Population and housing census, LISA		In quintiles per year/census. For births 1973-1979, we used census data from 1975. For births 1980-1987, census data from 1985. Births 1988-1990, LISA from 1990. For births 1991 onwards, LISA from the year before childbirth.
Maternal level of formal education	LISA		In year of childbirth, coded ≤9 years, 10-12 years, >12 years. Data only available from 1990, thus educational level in 1990 was used for those with earlier births.
Maternal inflammatory disease (SpA, IBD, psoriasis)	NPR	ICD-8 563.00, 563.10, 563.99, 569.02, 696, 712.40, 726.99<br>ICD-9 555, 556, 696, 713D, 720A, 720W, 720X, 713B 711B, 711D-711F, 711X<br>ICD-10 K50, M09.1, K51, M09.2 M08.1, M45.9, M46.8, M46.9, M07.2, M07.4, M07.5, L40, M07.3, M09.0, M02.3, M02.8, M02.9	Hospitalisation with any of the listed ICD codes before childbirth.
SpA manifestations			
Anterior uveitis	NPR	ICD-10 H20, H22.1	≥1 visit with a recorded diagnosis of anterior uveitis at a specialist clinic of ophthalmology 1997-2022.
Psoriasis	NPR, PDR	ICD-10 L40 (except L40.5)<br>ATC D05	≥1 visit with a recorded diagnosis of psoriasis at a specialist clinic of dermatology 1997-2022, or dispensed prescription of ATC D05 2005-2022.
IBD	NPR	ICD-10 K50, M07.4, M09.1 K51, M07.5, M09.2	≥1 visit with a recorded diagnosis of IBD at a specialist clinic of internal medicine, rheumatology or gastroenterology 1997-2022.
**SpA-free sibling**	MGR, MBR, NPR	ICD-8 563.00, 563.10, 563.99, 569.02, 696, 712.40, 726.99<br>ICD-9 555, 556, 696, 713D, 720A, 720W, 720X, 713B 711B, 711D-711F, 711X<br>ICD-10 K50, M09.1, K51, M09.2 M08.1, M45.9, M46.8, M46.9, M07.2, M07.4, M07.5, L40, M07.3, M09.0, M02.3, M02.8, M02.9	Any full sibling of an AS index case, whose birth is registered in MBR and who has NOT received a diagnosis of the listed ICD codes (of SpA, IBD, or psoriasis) in NPR at a specialist clinic of internal medicine or rheumatology 2001-2022.

ATC, Anatomic Therapeutic Chemical; ICD, International Classification of Diseases; NPR, National Patient Register; MBR, Medical Birth Register; MGR, Multi-Generation Register; LISA, Longitudinal integrated database for health insurance and labour market studies; PDR, Prescribed Drug Register; AS, ankylosing spondylitis; SpA, spondyloarthritis; IBD, inflammatory bowel disease.

Supplemental table 2. ICD codes used to identify infections during pregnancy and childhood, according to the different ICD editions

Years	ICD version	Diagnostic codes
**-1986**	ICD-8	000-136, 320, 322, 360.00, 366.00, 369.00, 380-383, 420, 421, 422, 460-466, 470-474, 480-486, 490, 500.99, 501, 503, 508.00-508.02, 510, 511.10, 513.99, 522.40, 522.50, 522.70, 527.30, 540-543, 562, 566, 567.00-567.02, 569.00, 572.99, 590, 595.00, 595.09, 597.00, 599.02, 611.00-611.01, 612-614, 616.00-616.03, 620, 622.00, 622.19, 629.40, 630, 635, 636, 645.90-645.91, 680-686, 710, 720, 732.99, 761.00, 761.20-761.40, 763.00, 763.10, 998.50, 999.30
**1987-1996**	ICD-9	001-139, 254B, 279K, 320, 321, 322, 324, 360A, 372A, 380B, 382, 383, 420X, 421, 422X, 460-466, 473, 474, 475, 480-487, 490, 510, 511B, 513, 522E, 522F, 522H, 526E, 527D, 540-543, 562, 566, 567B, 567C, 569F, 572A, 590B, 595A, 595X, 597A, 597W, 599A, 601C, 611A, 614A-F, 615A, 616, 639A, 646F, 646G, 647, 658E, 659D, 680-686, 711A, 728A, 730, 770A, 771, 996G, 998F, 999D
**1997-**	ICD-10*	A00-B99, E32.1, G00-02, G04.2, G05.0, G05-G07, H10.0, H13.0, H13.1, H44.0, H60.0–H60.3, H62.0-H62.3, H66, H67, H70, I30.1, I33.0, I40.0, J00-J22, J32, J34.0, J35, J36, J39.0, J39.1, J44.0, J85, J86, K04.4, K04.6, K04.7, K10.2, K11.3, K35-38, K57.0, K57.2, K57.4, K57.8, K61, K63.0, K65.0, K67, K75.0, L00-08, L30.3, M00-01, M46.2, M46.3, M46.5, M49.0-M49.3, M60.0, M65.0, M65.1, M71.0, M71.1, M72.6, M86, N10, N13.6, N15.1, N30.0, N30.9, N34.0, N34.1, N34.2, N39.0, N41.2, N61, N70, N71.0, N72, N73.0, N73.3, N75.1, N76.0, N76.2, N76.4, N76.8b, O07.0, O07.5, O08.0, O23, O41.1, O75.3, O98, P23, P35-37, T80.2, T81.4, T82.6, T82.7, T83.5, T83.6, T84.5-T84.7, T85.7, T88.0, Z21

*Skåne county used ICD-9 until 1997

ICD, International Classification of Diseases.

Supplemental table 3. Number and proportion of subjects with missing data

	**AS cases (N=5612)**	**Population controls (N=22,042)**
**Maternal smoking**	2799 (49.9)	10884 (49.4)
**Maternal smoking (births from 1982)***	289 (9.3)	1105 (9.0)
**Maternal BMI**	3551 (63.3)	14053 (63.8)
**Maternal BMI (births from 1982)***	1041 (33.6)	4274 (34.9)
**Birthweight**	17 (0.3)	50 (0.2)
**Gestational age**	14 (0.2)	65 (0.3)
**Weight for gestational age**	30 (0.5)	114 (0.5)
**Parental country of birth**	33 (0.6)	142 (0.6)
**Maternal disposable income**	54 (1.0)	212 (1.0)
**Maternal educational level**	71 (1.3)	327 (1.5)

Data are n (%). Only variables with missing data included.

*Data on maternal smoking and maternal BMI was not available for births before 1982. Total number of births 1982-2004 (and denominators for these proportions) are 3102 in AS cases and 12263 in population controls.

AS, ankylosing spondylitis; BMI, body-mass index.

Supplemental table 4. Odds ratios for AS from conditional logistic regression on 25 multiply imputed datasets

Risk factor	AS cases (N=5612)	Population controls (N=22,042)	OR (95% CI)	Adj. OR* (95% CI)
**Maternal age in years, mean (SD)**	27.4 (5.0)	27.5 (5.1)	1.00 (0.99 to 1.00)	1.00 (0.99 to 1.00)
**Maternal smoking†, n (%)**	687 (24.4)	2774 (24.9)	0.98 (0.89 to 1.08)	0.99 (0.90 to 1.10)
**Maternal BMI† in kg/m², mean (SD)¶**	22.6 (3.4)	22.8 (3.6)	0.99 (0.98 to 1.00)	0.99 (0.98 to 1.01)
<18.5 kg/m², n (%)	102 (4.9)	496 (6.2)	0.84 (0.68 to 1.04)	0.85 (0.68 to 1.05)
18.5-24.9 kg/m², n (%)	1570 (76.2)	5806 (72.7)	Reference	Reference
25-29.9 kg/m², n (%)	324 (15.7)	1330 (16.6)	0.93 (0.81 to 1.06)	0.93 (0.82 to 1.07)
≥30 kg/m², n (%)	65 (3.2)	357 (4.5)	0.80 (0.62 to 1.03)	0.80 (0.62 to 1.04)
**Gestational age, mean (SD)**	40 (2)	40 (2)	0.98 (0.97 to 1.00)	0.99 (0.97 to 1.00)
Very preterm, n (%)	29 (0.5)	106 (0.5)	1.08 (0.72 to 1.64)	1.07 (0.71 to 1.63)
Preterm, n (%)	283 (5.1)	1084 (4.9)	1.02 (0.89 to 1.17)	1.00 (0.87 to 1.15)
Term, n (%)	3776 (67.5)	14572 (66.3)	Reference	Reference
Post-term, n (%)	1510 (27.0)	6215 (28.3)	0.94 (0.88 to 1.00)	0.94 (0.88 to 1.01)
**Weight for gestational age in SDs, mean (SD)**	-0.10 (1.12)	-0.10 (1.10)	1.01 (0.98 to 1.03)	1.01 (0.98 to 1.04)
SGA (>2 SD below mean), n (%)	210 (3.8)	733 (3.3)	1.12 (0.96 to 1.31)	1.10 (0.94 to 1.30)
Moderately small (1-2 SD below), n (%)	885 (15.9)	3483 (15.9)	1.00 (0.92 to 1.08)	1.00 (0.92 to 1.08)
Normal (±1 SD), n (%)	3691 (66.1)	14582 (66.5)	Reference	Reference
Moderately large (1-2 SD above), n (%)	652 (11.7)	2493 (11.4)	1.04 (0.94 to 1.14)	1.04 (0.95 to 1.14)
LGA (>2 SD above mean), n (%)	144 (2.6)	637 (2.9)	0.90 (0.75 to 1.08)	0.91 (0.76 to 1.10)
**Birthweight in grams‡, mean (SD)**	3508 (563)	3513 (553)	1.00 (0.99 to 1.00)	1.00 (0.99 to 1.01)
<2500 g, n (%)	218 (3.9)	816 (3.7)	1.05 (0.90 to 1.23)	1.02 (0.87 to 1.20)
2500-4299 g, n (%)	4990 (89.2)	19679 (89.5)	Reference	Reference
≥4300 g, n (%)	387 (6.9)	1497 (6.8)	1.03 (0.91 to 1.16)	1.04 (0.92 to 1.17)
**Multiple birth, n (%)**	130 (2.3)	423 (1.9)	1.22 (1.00 to 1.49)	1.23 (1.01 to 1.50)
**Caesarean section, n (%)**	624 (11.1)	2313 (10.5)	1.07 (0.98 to 1.18)	1.07 (0.97 to 1.18)
**Maternal infection during pregnancy, n (%)**	134 (2.4)	477 (2.2)	1.09 (0.90 to 1.33)	1.08 (0.89 to 1.32)
**Season of birth, n (%)**				
Dec-Feb	1399 (24.9)	5170 (23.5)	Reference	Reference
March-May	1615 (28.8)	6128 (27.8)	0.97 (0.90 to 1.05)	0.97 (0.90 to 1.05)
June-Aug	1381 (24.6)	5584 (25.3)	0.91 (0.84 to 0.99)	0.91 (0.84 to 0.99)
Sep-Nov	1217 (21.7)	5160 (23.4)	0.87 (0.79 to 0.94)	0.87 (0.80 to 0.95)
**No. of full siblings in total, n (%)**				
0	881 (15.7)	3498 (15.9)	Reference	Reference
1	2557 (45.6)	9982 (45.3)	1.02 (0.94 to 1.12)	1.01 (0.92 to 1.11)
2 or more	2174 (38.7)	8562 (38.8)	1.02 (0.93 to 1.11)	1.01 (0.92 to 1.10)
**No. of older siblings, n (%)**				
0	2164 (38.6)	9098 (41.3)	Reference	Reference
1	2172 (38.7)	8081 (36.7)	1.13 (1.06 to 1.21)	1.12 (1.04 to 1.22)
2 or more	1276 (22.7)	4863 (22.1)	1.11 (1.03 to 1.20)	1.15 (1.04 to 1.27)
**No. of younger siblings, n (%)**				
0	3470 (61.8)	13155 (59.7)	Reference	Reference
1	1531 (27.3)	6245 (28.3)	0.93 (0.87 to 1.00)	0.97 (0.89 to 1.04)
2 or more	611 (10.9)	2642 (12.0)	0.88 (0.80 to 0.97)	0.91 (0.81 to 1.02)
**Serious childhood infection§, n (%)**	1396 (24.9)	5006 (22.8)	1.13 (1.05 to 1.21)	1.13 (1.05 to 1.21)
**Tonsillectomy§, n (%)**	279 (5.0)	855 (3.9)	1.29 (1.12 to 1.49)	1.30 (1.13 to 1.49)
**Appendectomy§, n (%)**	126 (2.3)	573 (2.6)	0.87 (0.71 to 1.06)	0.87 (0.71 to 1.06)

*Adjusted for maternal age, maternal BMI, maternal smoking, parental country of birth, maternal disposable income, maternal educational level, maternal inflammatory disease (hospitalisation for SpA, IBD, psoriasis), and multiple birth, with the exception that no exposure was adjusted for itself. Number of older siblings additionally adjusted for number of younger siblings, and vice versa.
†Analyses for smoking and BMI restricted to birth years from 1982 onwards.
‡Analysed per 100 g.
§Until age 15. 16 cases and their 72 matched controls excluded due to AS diagnosis in index case before age 16.
AS, ankylosing spondylitis; BMI, body-mass index; SpA, spondyloarthritis; IBD, inflammatory bowel disease.

Supplemental table 5. Odds ratios for AS from conditional logistic regression on AS cases and population controls with complete data on all variables

Risk factor	AS cases (N=1824)	Population controls (N=5317)	OR (95% CI)	Adj. OR* (95% CI)
**Maternal age in years, mean (SD)**	28.2 (5.0)	28.2 (5.1)	1.00 (0.99 to 1.01)	1.00 (0.99 to 1.01)
**Maternal smoking†, n (%)**	428 (23.4)	1297 (24.4)	0.94 (0.82 to 1.07)	0.94 (0.82 to 1.07)
**Maternal BMI† in kg/m², mean (SD)¶**	22.7 (3.4)	22.9 (3.6)	0.99 (0.97 to 1.00)	0.99 (0.97 to 1.00)
<18.5 kg/m², n (%)	87 (4.8)	312 (5.9)	0.75 (0.58 to 0.96)	0.75 (0.58 to 0.96)
18.5-24.9 kg/m², n (%)	1392 (76.2)	3828 (72.0)	Reference	Reference
25-29.9 kg/m², n (%)	287 (15.7)	925 (17.4)	0.89 (0.76 to 1.03)	0.89 (0.76 to 1.03)
≥30 kg/m², n (%)	61 (3.3)	252 (4.7)	0.70 (0.52 to 0.94)	0.70 (0.52 to 0.94)
**Gestational age, mean (SD)**	40 (2)	40 (2)	1.01 (0.98 to 1.04)	1.02 (0.99 to 1.05)
Very preterm, n (%)	5 (0.3)	19 (0.4)	0.75 (0.28 to 2.04)	0.70 (0.25 to 1.95)
Preterm, n (%)	85 (4.7)	287 (5.4)	0.85 (0.66 to 1.10)	0.81 (0.63 to 1.06)
Term, n (%)	1292 (70.7)	3699 (69.6)	Reference	Reference
Post-term, n (%)	445 (24.4)	1312 (24.7)	0.98 (0.86 to 1.11)	0.99 (0.87 to 1.12)
**Weight for gestational age in SDs, mean (SD)**	0.04 (1.04)	-0.00 (1.06)	1.05 (0.99 to 1.10)	1.05 (1.00 to 1.11)
SGA (>2 SD below mean), n (%)	44 (2.4)	132 (2.5)	0.99 (0.70 to 1.42)	0.99 (0.69 to 1.42)
Moderately small (1-2 SD below), n (%)	234 (12.8)	695 (13.1)	0.98 (0.83 to 1.16)	0.98 (0.83 to 1.16)
Normal (±1 SD), n (%)	1242 (68.0)	3681 (69.2)	Reference	Reference
Moderately large (1-2 SD above), n (%)	254 (13.9)	622 (11.7)	1.23 (1.04 to 1.45)	1.24 (1.05 to 1.46)
LGA (>2 SD above mean), n (%)	53 (2.9)	187 (3.5)	0.79 (0.57 to 1.09)	0.81 (0.59 to 1.13)
**Birthweight in grams‡, mean (SD)**	3556 (540)	3531 (549)	1.01 (1.00 to 1.02)	1.01 (1.00 to 1.02)
<2500 g, n (%)	56 (3.1)	190 (3.6)	0.87 (0.64 to 1.19)	0.81 (0.59 to 1.13)
2500-4299 g, n (%)	1627 (89.1)	4752 (89.4)	Reference	Reference
≥4300 g, n (%)	144 (7.9)	375 (7.1)	1.14 (0.93 to 1.39)	1.16 (0.94 to 1.43)
**Multiple birth, n (%)**	43 (2.4)	97 (1.8)	1.31 (0.90 to 1.89)	1.32 (0.91 to 1.91)
**Caesarean section, n (%)**	217 (11.9)	583 (11.0)	1.09 (0.92 to 1.30)	1.08 (0.90 to 1.28)
**Maternal infection during pregnancy, n (%)**	50 (2.7)	151 (2.8)	0.93 (0.67 to 1.29)	0.92 (0.66 to 1.29)
**Season of birth, n (%)**				
Dec-Feb	478 (26.2)	1217 (22.9)	Reference	Reference
March-May	516 (28.2)	1480 (27.8)	0.90 (0.77 to 1.04)	0.90 (0.77 to 1.04)
June-Aug	453 (24.8)	1384 (26.0)	0.84 (0.72 to 0.98)	0.83 (0.71 to 0.97)
Sep-Nov	380 (20.8)	1236 (23.2)	0.77 (0.65 to 0.90)	0.77 (0.65 to 0.90)
**No. of full siblings in total, n (%)**				
0	252 (13.8)	799 (15.0)	Reference	Reference
1	875 (47.9)	2466 (46.4)	1.13 (0.96 to 1.33)	1.12 (0.95 to 1.33)
2 or more	700 (38.3)	2052 (38.6)	1.06 (0.89 to 1.25)	1.04 (0.87 to 1.25)
**No. of older siblings, n (%)**				
0	715 (39.1)	2214 (41.6)	Reference	Reference
1	672 (36.8)	1929 (36.3)	1.09 (0.96 to 1.24)	1.12 (0.96 to 1.31)
2 or more	440 (24.1)	1174 (22.1)	1.17 (1.01 to 1.35)	1.24 (1.03 to 1.49)
**No. of younger siblings, n (%)**				
0	1090 (59.7)	3136 (59.0)	Reference	Reference
1	536 (29.3)	1556 (29.3)	0.98 (0.87 to 1.11)	1.05 (0.91 to 1.21)
2 or more	201 (11.0)	625 (11.8)	0.90 (0.75 to 1.07)	0.96 (0.78 to 1.18)
**Serious childhood infection§, n (%)**	518 (28.5)	1370 (25.9)	1.15 (1.02 to 1.30)	1.16 (1.02 to 1.31)
**Tonsillectomy§, n (%)**	104 (5.7)	261 (4.9)	1.15 (0.91 to 1.47)	1.17 (0.92 to 1.49)
**Appendectomy§, n (%)**	40 (2.2)	111 (2.1)	1.12 (0.77 to 1.62)	1.12 (0.77 to 1.63)

*Adjusted for maternal age, maternal BMI, maternal smoking, parental country of birth, maternal disposable income, maternal educational level, maternal inflammatory disease (hospitalisation for SpA, IBD, psoriasis), and multiple birth, with the exception that no exposure was adjusted for itself. Number of older siblings additionally adjusted for number of younger siblings, and vice versa.
†Analysed per 100 g.
‡Until age 15. 16 cases and their 72 matched controls excluded due to AS diagnosis in index case before age 16.
AS, ankylosing spondylitis; BMI, body-mass index; SpA, spondyloarthritis; IBD, inflammatory bowel disease.

Supplemental table 6. Odds ratios for AS in men and females separately, in relation to childhood infections and perinatal factors, with p-values for significance of interaction by sex

Risk factor	Females cases/controls	Females OR (95% CI)	Female Adj. OR* (95% CI)	Males cases/controls	Males OR (95% CI)	Males Adj. OR* (95% CI)	p-value for interaction
**Maternal age in years, mean (SD)**	27.3 (5.1)/27.4 (5.1)	1.00 (0.99 to 1.00)	1.00 (0.99 to 1.00)	27.5 (5.0)/27.5 (5.1)	1.00 (0.99 to 1.01)	1.00 (0.99 to 1.01)	0.8212
**Maternal smoking†, n (%)**	326 (27.2)/1158 (24.3)	1.15 (1.00 to 1.33)	**1.13 (0.97 to 1.32)**	361 (22.4)/1616 (25.3)	0.87 (0.76 to 0.99)	**0.89 (0.78 to 1.02)**	**0.0226**
**Maternal BMI† in kg/m², mean (SD)¶**	22.7 (3.4)/22.8 (3.6)	0.99 (0.97 to 1.01)	0.99 (0.97 to 1.01)	22.6 (3.4)/22.8 (3.6)	0.99 (0.97 to 1.01)	0.99 (0.97 to 1.01)	0.8196
<18.5 kg/m², n (%)	42 (4.9)/237 (6.9)	0.78 (0.55 to 1.10)	0.78 (0.55 to 1.10)	60 (5.0)/259 (5.7)	0.89 (0.68 to 1.18)	0.90 (0.68 to 1.19)	0.5145
18.5-24.9 kg/m², n (%)	657 (76.2)/2495 (72.7)	Reference	Reference	913 (76.1)/3311 (72.7)	Reference	Reference	
25-29.9 kg/m², n (%)	135 (15.7)/550 (16.0)	0.95 (0.77 to 1.16)	0.95 (0.78 to 1.16)	189 (15.8)/780 (17.1)	0.91 (0.77 to 1.08)	0.92 (0.78 to 1.10)	0.8402
≥30 kg/m², n (%)	28 (3.2)/152 (4.4)	0.79 (0.53 to 1.17)	0.79 (0.53 to 1.17)	37 (3.1)/205 (4.5)	0.80 (0.57 to 1.11)	0.81 (0.58 to 1.13)	0.9255
**Gestational age, mean (SD)**	40 (2)/40 (2)	0.98 (0.96 to 1.00)	0.98 (0.96 to 1.01)	40 (2)/40 (2)	0.99 (0.97 to 1.01)	0.99 (0.97 to 1.01)	0.6835
Very preterm, n (%)	11 (0.5)/52 (0.6)	0.84 (0.43 to 1.61)	0.82 (0.42 to 1.59)	18 (0.6)/54 (0.4)	1.32 (0.77 to 2.25)	1.34 (0.78 to 2.30)	0.2619
Preterm, n (%)	112 (4.7)/432 (4.6)	0.98 (0.79 to 1.21)	0.95 (0.76 to 1.19)	171 (5.4)/652 (5.2)	1.05 (0.88 to 1.25)	1.03 (0.87 to 1.24)	0.5764
Term, n (%)	1660 (68.9)/6272 (66.5)	Reference	Reference	2116 (66.3)/8300 (66.1)	Reference	Reference	
Post-term, n (%)	625 (26.0)/2673 (28.3)	0.88 (0.80 to 0.98)	0.88 (0.80 to 0.98)	885 (27.7)/3542 (28.2)	0.98 (0.90 to 1.07)	0.98 (0.90 to 1.08)	0.1241
**Weight for gestational age in SDs, mean (SD)**	-0.16 (1.07)/-0.13 (1.08)	0.97 (0.93 to 1.01)	**0.98 (0.94 to 1.02)**	-0.05 (1.09)/-0.08 (1.07)	1.03 (1.00 to 1.07)	**1.04 (1.00 to 1.08)**	**0.0341**
SGA (>2 SD below mean), n (%)	97 (4.0)/326 (3.5)	1.15 (0.91 to 1.45)	1.12 (0.88 to 1.42)	113 (3.6)/407 (3.2)	1.09 (0.88 to 1.36)	1.09 (0.88 to 1.36)	0.8635
Moderately small (1-2 SD below), n (%)	405 (16.9)/1526 (16.2)	1.03 (0.91 to 1.17)	1.03 (0.91 to 1.17)	480 (15.1)/1957 (15.6)	0.97 (0.87 to 1.08)	0.97 (0.87 to 1.09)	0.4936
Normal (±1 SD), n (%)	1596 (66.5)/6268 (66.7)	Reference	Reference	2095 (65.8)/8314 (66.4)	Reference	Reference	
Moderately large (1-2 SD above), n (%)	252 (10.5)/1014 (10.8)	0.98 (0.85 to 1.14)	0.99 (0.85 to 1.15)	400 (12.6)/1479 (11.8)	1.07 (0.95 to 1.21)	1.08 (0.95 to 1.22)	0.3831
LGA (>2 SD above mean), n (%)	49 (2.0)/268 (2.9)	0.73 (0.53 to 0.99)	0.73 (0.54 to 1.00)	95 (3.0)/369 (2.9)	1.03 (0.82 to 1.30)	1.04 (0.83 to 1.31)	0.0760
**Birthweight in grams‡, mean (SD)**	3422 (542)/3441 (539)	0.99 (0.98 to 1.00)	0.99 (0.99 to 1.00)	3573 (570)/3566 (558)	1.00 (1.00 to 1.01)	1.00 (1.00 to 1.01)	0.1257
<2500 g, n (%)	112 (4.7)/385 (4.1)	1.13 (0.91 to 1.41)	1.10 (0.87 to 1.37)	106 (3.3)/431 (3.4)	0.98 (0.79 to 1.22)	0.95 (0.76 to 1.20)	0.3958
2500-4299 g, n (%)	2184 (90.9)/8584 (91.1)	Reference	Reference	2806 (87.9)/11095 (88.3)	Reference	Reference	
≥4300 g, n (%)	106 (4.4)/454 (4.8)	0.91 (0.73 to 1.13)	0.92 (0.74 to 1.15)	281 (8.8)/1043 (8.3)	1.08 (0.94 to 1.24)	1.09 (0.95 to 1.25)	0.2078
**Multiple birth, n (%)**	57 (2.4)/195 (2.1)	1.17 (0.87 to 1.58)	1.18 (0.87 to 1.59)	73 (2.3)/228 (1.8)	1.27 (0.97 to 1.66)	1.28 (0.98 to 1.67)	0.6830

Risk factor cont.	Females cases/controls	Females OR (95% CI)	Female Adj. OR* (95% CI)	Males cases/controls	Males OR (95% CI)	Males Adj. OR* (95% CI)	p-value for interaction
**Caesarean section, n (%)**	277 (11.5)/972 (10.3)	1.13 (0.98 to 1.31)	1.13 (0.98 to 1.31)	347 (10.8)/1341 (10.6)	1.03 (0.91 to 1.17)	1.02 (0.90 to 1.16)	0.2862
**Maternal infection during pregnancy, n (%)**	57 (2.4)/198 (2.1)	1.15 (0.85 to 1.55)	1.13 (0.83 to 1.52)	77 (2.4)/279 (2.2)	1.05 (0.81 to 1.36)	1.05 (0.81 to 1.36)	0.7302
**Season of birth, n (%)**							
Dec-Feb	587 (24.3)/2184 (23.1)	Reference	Reference	812 (25.4)/2986 (23.7)	Reference	Reference	
March-May	711 (29.5)/2626 (27.8)	1.00 (0.89 to 1.14)	1.00 (0.88 to 1.14)	904 (28.2)/3502 (27.8)	0.95 (0.85 to 1.06)	0.95 (0.85 to 1.05)	0.5022
June-Aug	584 (24.2)/2453 (26.0)	0.88 (0.77 to 1.00)	0.88 (0.77 to 1.00)	797 (24.9)/3131 (24.9)	0.93 (0.84 to 1.04)	0.93 (0.83 to 1.04)	0.5046
Sep-Nov	529 (21.9)/2187 (23.1)	0.89 (0.78 to 1.02)	0.89 (0.78 to 1.02)	688 (21.5)/2973 (23.6)	0.85 (0.76 to 0.95)	0.85 (0.76 to 0.95)	0.5817
**No. of full siblings in total, n (%)**							
0	414 (17.2)/1496 (15.8)	Reference	Reference	467 (14.6)/2002 (15.9)	Reference	Reference	
1	1059 (43.9)/4329 (45.8)	0.89 (0.78 to 1.01)	**0.90 (0.78 to 1.03)**	1498 (46.8)/5653 (44.9)	1.15 (1.02 to 1.29)	**1.11 (0.98 to 1.25)**	**0.0222**
2 or more	938 (38.9)/3625 (38.4)	0.94 (0.82 to 1.07)	0.96 (0.84 to 1.10)	1236 (38.6)/4937 (39.2)	1.09 (0.97 to 1.23)	1.05 (0.92 to 1.19)	0.3629
**No. of older siblings, n (%)**							
0	925 (38.4)/3967 (42.0)	Reference	Reference	1239 (38.7)/5131 (40.7)	Reference	Reference	
1	908 (37.7)/3407 (36.1)	1.14 (1.02 to 1.26)	1.13 (0.99 to 1.27)	1264 (39.5)/4674 (37.1)	1.12 (1.03 to 1.23)	1.12 (1.01 to 1.25)	0.9782
2 or more	578 (24.0)/2076 (22.0)	1.19 (1.06 to 1.34)	1.24 (1.07 to 1.44)	698 (21.8)/2787 (22.1)	1.05 (0.95 to 1.17)	1.09 (0.95 to 1.25)	0.2064
**No. of younger siblings, n (%)**							
0	1524 (63.2)/5605 (59.3)	Reference	Reference	1946 (60.8)/7550 (60.0)	Reference	Reference	
1	639 (26.5)/2710 (28.7)	0.88 (0.79 to 0.97)	0.91 (0.80 to 1.03)	892 (27.9)/3535 (28.1)	0.98 (0.89 to 1.07)	1.01 (0.91 to 1.12)	0.2097
2 or more	248 (10.3)/1135 (12.0)	0.81 (0.70 to 0.94)	0.84 (0.71 to 1.00)	363 (11.3)/1507 (12.0)	0.93 (0.82 to 1.05)	0.96 (0.83 to 1.11)	0.2275
**Serious childhood infection§, n (%)**	590 (24.5)/1935 (20.5)	1.27 (1.14 to 1.41)	**1.26 (1.13 to 1.40)**	806 (25.3)/3071 (24.5)	1.03 (0.94 to 1.13)	**1.04 (0.95 to 1.14)**	**0.0078**
**Tonsillectomy§, n (%)**	121 (5.0)/356 (3.8)	1.34 (1.08 to 1.66)	1.32 (1.06 to 1.64)	158 (5.0)/499 (4.0)	1.26 (1.05 to 1.52)	1.27 (1.06 to 1.54)	0.8094
**Appendectomy§, n (%)**	65 (2.7)/238 (2.5)	1.08 (0.82 to 1.43)	**1.08 (0.81 to 1.43)**	61 (1.9)/335 (2.7)	0.72 (0.55 to 0.95)	**0.72 (0.55 to 0.95)**	**0.0453**

Odds ratios from conditional logistic regression on 25 multiply imputed datasets, in AS cases and population controls stratified by sex.

*Adjusted for maternal age, maternal BMI, maternal smoking, parental country of birth, maternal disposable income, maternal educational level, maternal inflammatory disease (hospitalisation for SpA, IBD, psoriasis), and multiple birth, with the exception that no exposure was adjusted for itself. Number of older siblings additionally adjusted for number of younger siblings, and vice versa.

†Analyses for smoking and BMI restricted to birth years 1982-2004.

‡Analysed per 100 g.

§Until age 15.

AS, ankylosing spondylitis; BMI, body-mass index; SpA, spondyloarthritis; IBD, inflammatory bowel disease.

Supplemental table 7. Odds ratios for AS from sibling comparison analysis, only including individuals who reached age 35 during follow-up

Risk factor	AS cases (N=2317)	SpA-free siblings (N=3193)	OR (95% CI)	Adj. OR* (95% CI)
**Multiple birth, n (%)**	80 (3.5)	110 (3.4)	1.46 (0.86 to 2.50)	1.36 (0.79 to 2.33)
**Season of birth, n (%)**				
Dec-Feb	585 (25.2)	779 (24.4)	Reference	Reference
March-May	656 (28.3)	917 (28.7)	0.96 (0.82 to 1.11)	0.97 (0.83 to 1.13)
June-Aug	565 (24.4)	792 (24.8)	0.97 (0.83 to 1.14)	0.96 (0.82 to 1.13)
Sep-Nov	511 (22.1)	705 (22.1)	1.00 (0.85 to 1.17)	1.00 (0.83 to 1.19)
**No. of older siblings, n (%)**				
0	782 (33.8)	1071 (33.5)	Reference	Reference
1	981 (42.3)	1145 (35.9)	1.09 (0.99 to 1.20)	1.35 (1.16 to 1.57)
2 or more	554 (23.9)	977 (30.6)	1.06 (0.91 to 1.22)	1.75 (1.31 to 2.34)
**Serious childhood infection†, n (%)**	536 (23.2)	692 (21.7)	1.08 (0.94 to 1.24)	1.07 (0.93 to 1.24)
**Tonsillectomy†, n (%)**	99 (4.3)	101 (3.2)	1.41 (1.04 to 1.92)	1.44 (1.06 to 1.96)

Odds ratios from conditional logistic regression.
*Adjusted for maternal age, year of birth, sex of child, and parity (except when exposure is number of older siblings).
†Until age 15. 3 cases and their 4 SpA-free siblings excluded due to AS diagnosis in index case before age 16.
AS, ankylosing spondylitis; SpA, spondyloarthritis.

Supplemental table 8. Odds ratios for AS only including cases and population controls who reached age 35 during follow-up

Risk factor	AS cases (N=3836)	Population controls (N=14,791)	OR (95% CI)	Adj. OR* (95% CI)
**Multiple birth, n (%)**	95 (2.5)	247 (1.7)	1.50 (1.18 to 1.90)	1.51 (1.18 to 1.92)
**Season of birth, n (%)**				
Dec-Feb	947 (24.7)	3405 (23.0)	Reference	Reference
March-May	1105 (28.8)	4171 (28.2)	0.95 (0.86 to 1.05)	0.96 (0.87 to 1.06)
June-Aug	940 (24.5)	3718 (25.1)	0.91 (0.82 to 1.00)	0.91 (0.82 to 1.01)
Sep-Nov	844 (22.0)	3497 (23.6)	0.86 (0.78 to 0.96)	0.87 (0.78 to 0.96)
**No. of older siblings, n (%)**				
0	1470 (38.3)	6134 (41.5)	Reference	Reference
1	1503 (39.2)	5486 (37.1)	1.14 (1.05 to 1.24)	1.13 (1.03 to 1.25)
2 or more	863 (22.5)	3171 (21.4)	1.14 (1.04 to 1.26)	1.18 (1.05 to 1.34)
**Serious childhood infection†, n (%)**	899 (23.5)	3227 (21.8)	1.09 (1.00 to 1.19)	1.09 (1.00 to 1.18)
**Tonsillectomy†, n (%)**	170 (4.4)	536 (3.6)	1.21 (1.01 to 1.45)	1.21 (1.01 to 1.45)

Odds ratios from conditional logistic regression on 25 multiply imputed datasets.
*Adjusted for maternal age, maternal BMI, maternal smoking, parental country of birth, maternal disposable income, maternal educational level, maternal inflammatory disease (hospitalisation for SpA, IBD, psoriasis), and multiple birth, with the exception that no exposure was adjusted for itself. Number of older siblings additionally adjusted for number of younger siblings.
†Until age 15. 3 cases and their 13 matched controls excluded due to AS diagnosis before age 16.
AS, ankylosing spondylitis.

Supplemental table 9. Odds ratios for AS in cases and population controls with at least one sibling

Risk factor	AS cases (N=4704)	Population controls (N=15,667)	OR (95% CI)	Adj. OR* (95% CI)
**Multiple birth, n (%)**	129 (2.7)	364 (2.3)	1.20 (0.98 to 1.47)	1.21 (0.98 to 1.48)
**Season of birth, n (%)**				
Dec-Feb	1161 (24.7)	3596 (23.0)	Reference	Reference
March-May	1373 (29.2)	4397 (28.1)	0.96 (0.87 to 1.05)	0.95 (0.87 to 1.04)
June-Aug	1158 (24.6)	3996 (25.5)	0.89 (0.81 to 0.98)	0.89 (0.81 to 0.98)
Sep-Nov	1012 (21.5)	3678 (23.5)	0.85 (0.77 to 0.93)	0.85 (0.77 to 0.94)
**No. of older siblings, n (%)**				
0	1564 (33.2)	5687 (36.3)	Reference	Reference
1	1997 (42.5)	6295 (40.2)	1.15 (1.07 to 1.24)	1.15 (1.03 to 1.29)
2 or more	1143 (24.3)	3685 (23.5)	1.13 (1.03 to 1.23)	1.18 (1.03 to 1.35)
**Serious childhood infection†, n (%)**	1159 (24.7)	3469 (22.2)	1.15 (1.06 to 1.24)	1.15 (1.06 to 1.24)
**Tonsillectomy†, n (%)**	236 (5.0)	611 (3.9)	1.31 (1.12 to 1.53)	1.32 (1.12 to 1.54)

Odds ratios from conditional logistic regression on 25 multiply imputed datasets.

*Adjusted for maternal age, maternal BMI, maternal smoking, parental country of birth, maternal disposable income, maternal educational level, maternal inflammatory disease (hospitalisation for SpA, IBD, psoriasis), and multiple birth, with the exception that no exposure was adjusted for itself. Number of older siblings additionally adjusted for number of younger siblings.

†Until age 15. 14 cases and their 51 matched controls excluded due to AS diagnosis in index case before age 16.

AS, ankylosing spondylitis.

Supplemental table 10. Odds ratios for AS in cases and population controls by timing of infection or procedure

	AS cases (N=5612)	Population controls (N=22,042)	OR (95% CI)
**Serious infection during first year of life, n (%)**	471 (8.4)	1721 (7.8)	1.08 (0.97 to 1.21)
**Tonsillectomy*, n (%)**			
No	5317 (95.0)	21115 (96.1)	Reference
<5 years of age	83 (1.5)	278 (1.3)	1.18 (0.92 to 1.51)
5-9 years	128 (2.3)	361 (1.6)	1.42 (1.15 to 1.74)
10-15 years	68 (1.2)	216 (1.0)	1.23 (0.93 to 1.63)
**Appendectomy*, n (%)**			
No	5470 (97.7)	21397 (97.4)	Reference
<5 years of age	6 (0.1)	32 (0.1)	0.70 (0.29 to 1.69)
5-9 years	30 (0.5)	185 (0.8)	0.62 (0.42 to 0.92)
10-15 years	90 (1.6)	356 (1.6)	1.02 (0.81 to 1.29)

Odds ratios from conditional logistic regression on 25 multiply imputed datasets.

*Until age 15. 16 cases and their 72 matched controls excluded due to AS diagnosis in index case before age 16.

AS, ankylosing spondylitis.
